# Change Detection of Optical Remote Sensing Image Disturbed by Thin Cloud Using Wavelet Coefficient Substitution Algorithm

**DOI:** 10.3390/s19091972

**Published:** 2019-04-26

**Authors:** Xiaoqian Yang, Zhenhong Jia, Jie Yang, Nikola Kasabov

**Affiliations:** 1 College of Information Science and Engineering, Xinjiang University, Urumuqi 830046, China; m15099106737@163.com; 2 Institute of Image Processing and Pattern Recognition, Shanghai Jiao Tong University, Shanghai 200240, China; Jieyang@sjtu.edu.cn; 3 Knowledge Engineering and Discovery Research Institute, Auckland University of Technology, 1020 Auckland, New Zealand; nkasabov@aut.ac.nz

**Keywords:** optical remote sensing image, thin cloud removal, combination difference map, FCM clustering, unsupervised, change detection

## Abstract

The detection of changes in optical remote sensing images under the interference of thin clouds is studied for the first time in this paper. First, the optical remote sensing image is subjected to thin cloud removal processing, and then the processed remote sensing image is subjected to image change detection. Based on the analysis of the characteristics of thin cloud images, a method for removing thin clouds based on wavelet coefficient substitution is proposed in this paper. Based on the change in the wavelet coefficient, the high- and low-frequency parts of the remote sensing image are replaced separately, and the low-frequency clouds are suppressed while maintaining the high-frequency detail of the image, which achieves good results. Then, an unsupervised change detection algorithm based on a combined difference graph and fuzzy c-means clustering algorithm (FCM) clustering is applied. First, the image is transformed into a logarithmic domain, and the image is denoised using Frost filtering. Then, the mean ratio method and the difference method are used to obtain two graph difference maps, and the combined difference graph method is used to obtain the final difference image. The experimental results show that the algorithm can effectively solve the problem of image change detection under thin cloud interference.

## 1. Introduction

Image change detection refers to the detection of landform changes in remote sensing images obtained at different times in the same area. Recently, with the development of remote sensing technology, optical remote sensing image change detection has been widely used in the fields of environmental monitoring, crop measurement, urban research, ecosystem monitoring, natural disaster assessment, battlefield target strike effect evaluation and military reconnaissance [1,2,3,4,5]. There are many algorithms for remote sensing image change detection [6,7,8,9,10,11]. Image change detection is mainly divided into pixel level change detection, feature level change detection and target level change detection [12,13]. Since most of the images acquired by remote sensing satellites are optical remote sensing images, such images are easily affected by severe weather, especially by clouds and fog. Cloud coverage is one of the important factors causing the lack of optical remote sensing data, which has an impact on the detection of changes in optical remote sensing images. Therefore, it is of great practical significance to study the change detection of optical remote sensing images under the condition of thin cloud interference.

In order to solve the problem that optical remote sensing images are affected by thin clouds, a new cloud removal algorithm combined with an image change detection method is proposed in this paper. Firstly, thin clouds are removed from optical remote sensing images, and then changes of remote sensing images after removing thin clouds are detected.

A large number of studies have shown that the thin cloud region on optical remote sensing images contains not only thin cloud information but also ground scene information. Through the removal of thin clouds, details of objects covered by thin clouds in optical remote sensing images can be recovered, which provides theoretical support for the removal of thin clouds and the detection of optical remote sensing image changes under the interference of clouds [14].

Scholars at home and abroad have attempted to remove cloud interference from optical remote sensing images by various methods to obtain clearer and more effective remote sensing images. For example, in [15,16], Wenting Cai and Zhou Xiao-Jun proposed improving the homomorphic filtering algorithm and designed a new homomorphic filter so that cloud-free area information could be recovered better, but the overall cloud removal was not ideal. In [17,18], Liu Yang and Lin Xiaojun propose an improved retinex theory, which overcomes the disadvantages of halos and poor effects on brighter image processing. However, the average grayscale of the result is relatively large. In [19], He Hui proposed an algorithm combining SWT (static wavelet transform) and DWT (discrete wavelet transforma) to improve the spatial resolution of the image, outstanding landscape characteristics and recovery. However, because the algorithm is on the high-frequency part of the SWT and DWT processing, it does not suppress the images with low frequency, leading to an increased average gray value of the image after processing, affecting the overall effect of the cloud. In [20,21], Xifang Zhou and Liu Yun proposed the Mallat algorithm to increase the low-level detail coefficient, highlight the scene information, reduce the high-level detail coefficient, and appropriately reduce the approximate coefficient to restrain thin clouds. However, the loss of ground object details is serious because it reduces the integrity of cloud removal. These methods have achieved good results for specific experimental data, but currently, there is no universal method.

A wavelet coefficient replacement algorithm for the removal of thin clouds is proposed in this paper. From the perspective of enhancing the contrast between low-frequency thin clouds and high-frequency ground details, the noise of thin clouds in optical remote sensing images is eliminated. In this paper, 80 groups of experiments were carried out to remove the thin cloud. The experimental results of the three groups (two real cloud images and one artificial simulation with added thin clouds) show that the method can effectively suppress the interference of optical remote image cloud noise and enhance the useful details.

Since the image has different degrees of noise interference after removing the thin cloud, in order to improve the detection accuracy of optical remote sensing images, an unsupervised change detection algorithm based on a combination of the difference image and FCM (fuzzy c-means clustering algorithm) clustering is proposed in this paper. To obtain the difference images with well-preserved change information before and after the removal of thin clouds, the image was first transformed into a number domain by the logarithmic transformation algorithm, and the noise was removed by Frost filtering [22]. Then, the mean ratio method [23] and the difference method [24] were used to obtain the difference image of the two images. Different denoising processes were carried out according to the noise characteristics of the two images, and the final difference image was obtained by combining the difference images. Finally, the FCM [25,26,27,28] clustering algorithm was used to divide the difference map into changed regions and non-changing regions. The experimental results show that the algorithm greatly reduces the difficulty of optical remote sensing image change detection under cloud interference and improves the detection accuracy.

## 2. Thin Cloud Removal Section

### 2.1. Thin Cloud Imaging Model and Thin Cloud Removal Difficulty Analysis

From the perspective of remote sensing physics, clouds belong to the category of atmospheric aerosols. They are a mixture of liquid ions or solid ions with a certain stability, small sedimentation velocity and scale between 10^−3^~10 μm in the Earth’s atmosphere. From the reflection characteristics of the cloud layer, the cloud layer in the visible light band is a diffuse reflection object. A large area of thin cloud frequently appears on optical remote sensing images. The image of the area covered by a thin cloud not only contains cloud information but also ground scenery information. At this time, the cloud exhibits a slowly changing airspace trend and has a low-frequency characteristic in the frequency domain of the image; compared with the thin cloud, the thick cloud occlusion image does not contain any information of the ground scene. When the clouds are thin, the imaging model of the scanner on the remote sensing satellite is shown in [29]. The image received on the scanner consists of two parts: the sunlight reflected through the cloud and the sunlight reflected from the ground and then penetrating the cloud.

(1) S(x,y)=ϕ[Lr(x,y)]=aLr(x,y)t(x,y)+L[1−t(x,y)].

*S* (*x*, *y*) is an image received by the image receiving equipment; *r* (*x*, *y*) is the reflectivity of the ground scenery representing the signal; *t* (*x*, *y*) is the transmittance of the cloud representing the noise; *L* is the intensity of the sunlight; *a* is the attenuation coefficient of the sunlight in the atmospheric transmission process, and the values of *r* (*x*, *y*), *t* (*x*, *y*) and *a* are between 0 and 1.

In the process of remote sensing image processing and analysis, the cloud-containing region of the remote sensing image has a local energy concentration in the low-frequency range. The removal of thin clouds is a difficult task in remote sensing image processing. As cloud obscurity prevents the image receiving equipment from accepting detailed information from the surface, the satellite cannot obtain effective cloud cover area information, and this area becomes a “blind area” in the image. The large area of the cloud layer occlusion seriously affects the quality of the image. How to completely remove the thin cloud in the remote sensing image is a problem that cannot be underestimated.

The method in this paper aims at the thin cloud which can see the information of ground scenery through the cloud layer subjectively. Due to the uneven thickness of the cloud layer, there is no critical cloud optical depth beyond which the algorithm cannot provide surface estimation. The experimental results showed that with the increase of cloud thickness, the subjective effect and objective evaluation index of thin cloud removal decrease. When the ground details are not visible through clouds, the effect of removing clouds by algorithm is not good, which will lead to the accuracy of subsequent image change detection.

### 2.2. Method for Removing Thin Clouds Based on Wavelet Coefficient Replacement

In view of the problems of the above methods, according to the wavelet decomposition principle [30], the image is subjected to multilevel two-dimensional discrete wavelet transformation, which can decompose the image into the low-frequency subband of the image approximation signal and the high-frequency subband of the image detail signal [31,32,33]. Among the subbands, most of the edge background details in the image belong to the high-frequency subband, while the low-frequency subband mainly represents the approximate signal of the image. The wavelet expansion is:

(2) $$f(t)=\sum_{k}c_{j.k}\phi_{j.k}(t)+\sum_{j}\sum_{k}d_{j.k}\varphi_{j,k}(t)$$

the first term on the right is the projection of *f*(*t*) on the scale space, which is a smooth approximation of *f*(*t*), and the second term is the projection of *f*(*t*) on the wavelet space, which is a supplement to the details of *f*(*t*). The decomposition form of *f*(*t*) is as follows:

(3) $$c_{j,k}=\langle f(x),\phi_{j,k}(x)\rangle =\int f(x)\phi_{j,k}{}_{}(x)dx$$

(4) $$d_{j,k}=\langle f(x),\varphi_{j,k}(x)\rangle =\int f(x)\varphi_{j,k}(x)dx$$

where $c_{j,k}$ is the approximation coefficient and $d_{j,k}$ is the detail coefficient.

Since the thin clouds are mainly concentrated in the low-frequency part of the image, the cloudless area is mainly concentrated in the high-frequency part of the image. To enhance the detailed information while reducing the influence of the thin clouds, the image is decomposed by wavelet decomposition, and the different frequency bands of the decomposed image are processed in a targeted manner while suppressing low-frequency thin clouds and enhancing the high-frequency details.

### 2.3. Algorithm Description

The general idea of thin cloud removal in this paper is to reduce the contrast of the high- and low-frequency parts of the image to achieve the purpose of highlighting the detailed object information and suppressing the low-frequency thin clouds; the innovation of the algorithm lies in the telescopic process of the wavelet coefficients and the change the wavelet coefficients use as the judgment conditions to separately replace the high and low frequencies of the image, and finally, the processed image is enhanced.

Wavelet decomposition can achieve the separation of the high-frequency components corresponding to the low-frequency components corresponding to the thin cloud cover and the scene details. To avoid the loss of the high-frequency detail part of the thin cloud, the high- and low-frequency parts can effectively solve this problem separately. Using the change of the wavelet coefficient after stretching as the condition, the part to be replaced is determined by the increase or decrease in the wavelet coefficients before and after telescopic processing. Thus, the image after replacement substantially increases the contrast of the source signal. The effect of thin cloud cover on the image can be regarded as the contrast decrease. Therefore, the image replacement with the wavelet coefficient as the judgment condition is expected to maintain the detailed information of the image and suppress the influence of the thin cloud cover. To obtain better details of the overall image, this paper’s image enhancement processing is performed on the basis of wavelet transform processing.

#### 2.3.1. Algorithm Implementation

First, the wavelet coefficients are telescopically processed; that is, the low-frequency part is linearly attenuated, and the high-frequency part is linearly enhanced. After the telescopic process of high- and low-frequency coefficient treatment, the wavelet coefficients in the cloud area decrease, and the wavelet coefficients in the cloudless area increase. Therefore, based on the wavelet decomposition coefficients, the telescopic image can be processed. In essence, the method of wavelet coefficient substitution is adopted. The original image and the processed image are replaced by the wavelet coefficients.

The telescopic process is as follows: the low-frequency part C_j,k_ = l × C_j,k_; the high-frequency part d_j,k_ = h × d_j,k_; where l ∈ (0, 1), h ∈ (1, 2); and l, h are the low-frequency cloud and high-frequency ground detail linear expansion coefficients, respectively.

The replacement process is as follows. Let the image represented by any pixel corresponding to the original image and the processed image be raw (*i*, *j*), after (*i*, *j*); let the wavelet coefficients of any pixel corresponding to the original image and the processed image be c_0_ (*i*, *j*), c_1_ (*i*, *j*); and let the image processed by the pixel replacement method be *F*(*i*, *j*). Then, there are:

(5) $$F(i,j)=\{\begin{array}{ll} raw(i,j); & c_{1}(i,j)>c_{0}(i,j)\\ after(i,j); & c_{1}(i,j)<=c_{0}(i,j) \end{array}$$

That is, when the processed wavelet coefficient increases, this area is a cloudless area and is replaced with the original image. When the processed wavelet coefficient is reduced, it indicates that this area is a cloud area and is replaced by a processed image.

#### 2.3.2. Algorithm Flowchart

**Figure 1:**
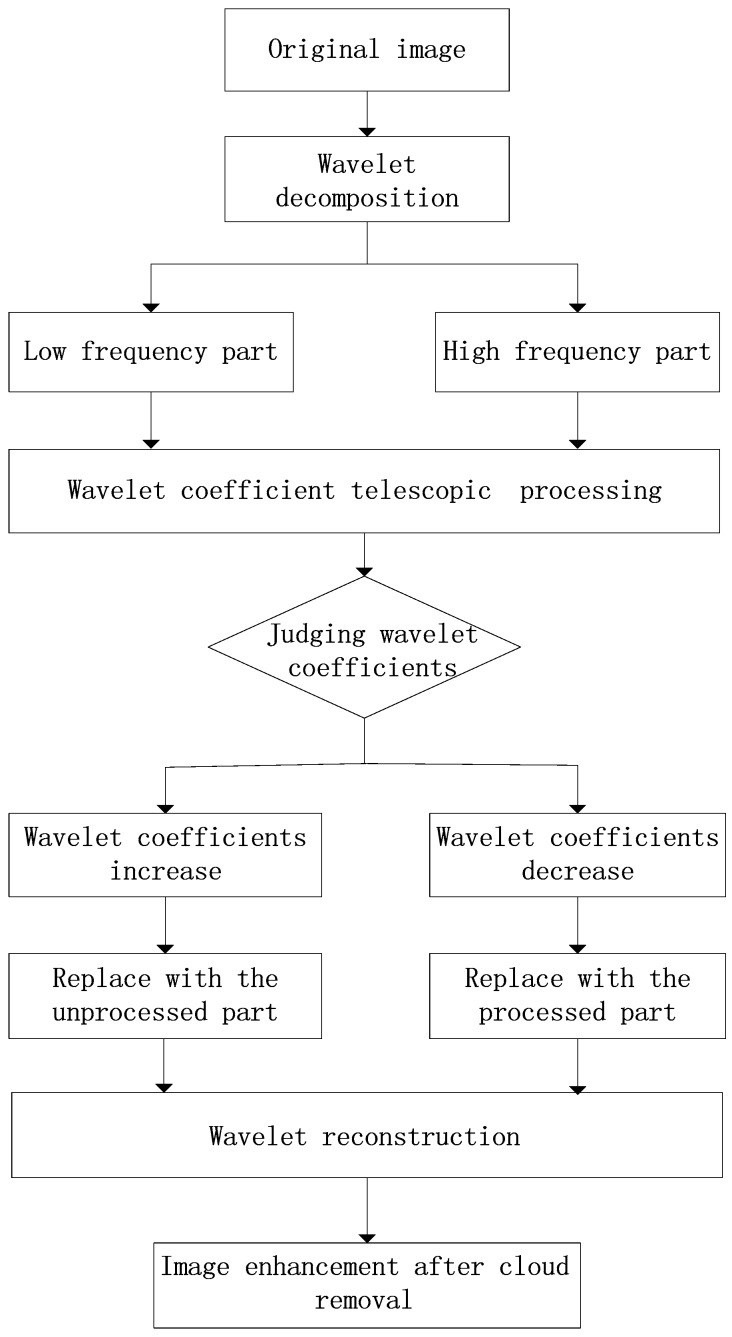
Thin cloud removal algorithm.

Figure 1 shows the thin cloud removal algorithm.

### 2.4. Experiments

**Figure 2:**
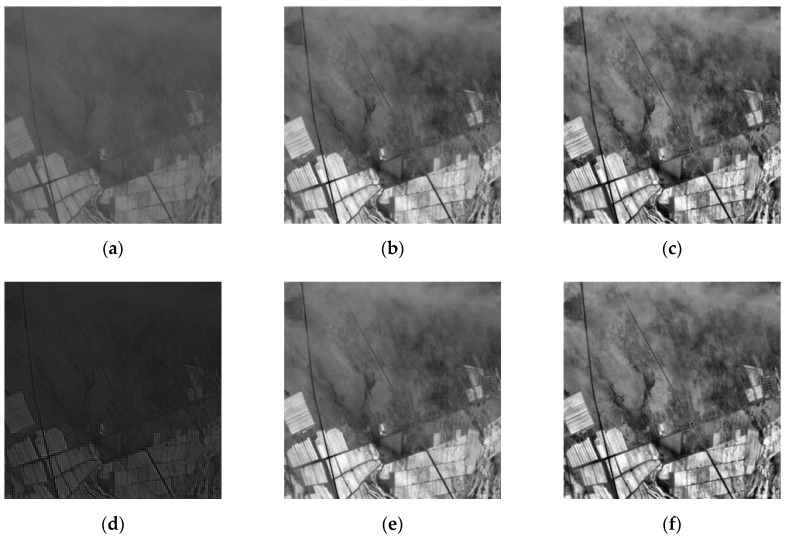
Comparison of different algorithms to clouds: (**a**) original image (real cloud image 1); (**b**) improved homomorphic filtering algorithm (IHF); (**c**) improved retinex algorithm (IR); (**d**) SWT + DWT algorithm (SD); (**e**) Mallat algorithm (M); (**f**) proposed method.

**Figure 3:**
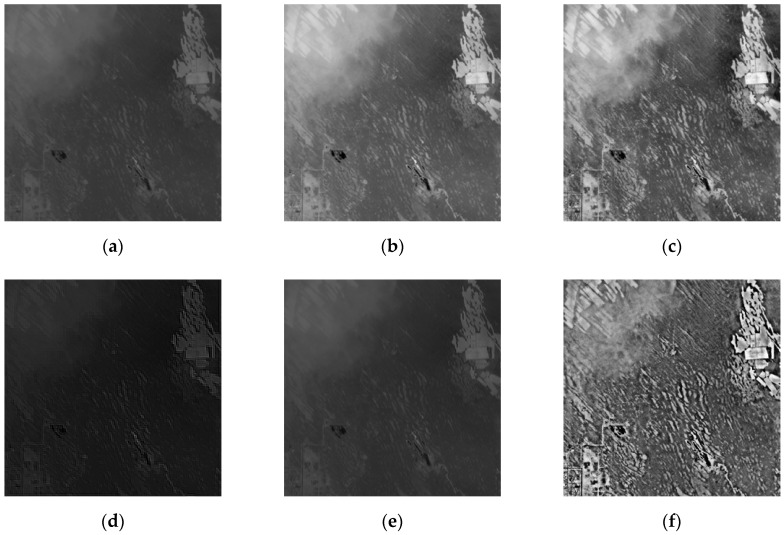
Comparison of different algorithms to cloud: (**a**) original image (real cloud image 2); (**b**) improved homomorphic filtering algorithm (IHF); (**c**) improved retinex algorithm (IR); (**d**) SWT + DWT algorithm (SD); (**e**) Mallat algorithm (M); (**f**) proposed method.

**Figure 4:**
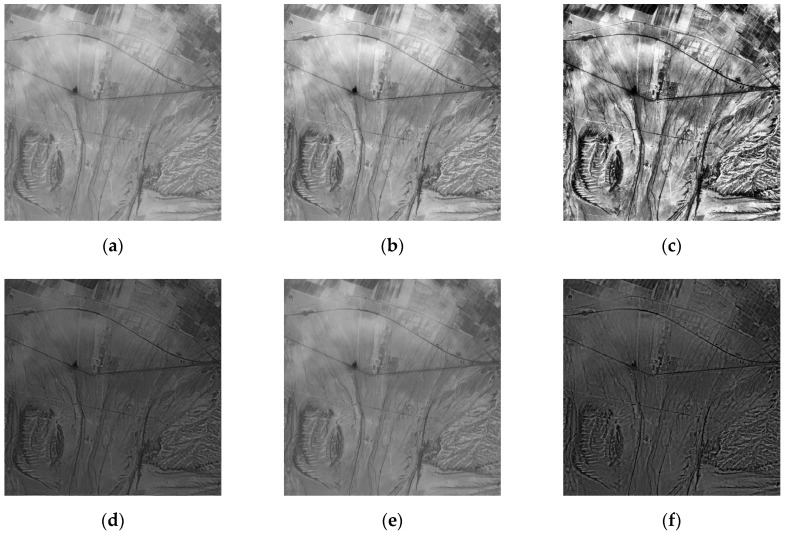
Comparison of different algorithms to clouds: (**a**) original image (artificial simulation to add clouds); (**b**) improved homomorphic filtering algorithm (IHF); (**c**) improved retinex algorithm (IR); (**d**) SWT + DWT algorithm (SD); (**e**) Mallat algorithm (M); (**f**) proposed method.

In this paper, three sets of optical remote sensing images seriously affected by thin clouds are selected. Figure 2 and Figure 3 are real optical remote sensing image data sets. The optical remote sensing images of the northern Xinjiang region were acquired through Landsat8 in July 2013 and July 2016 respectively, with the grayscale of 256 and the size of 280 × 280, 300 × 300. Figure 4 is the optical remote sensing image data set of artificially simulated thin cloud. The optical remote sensing image of Changji region in Xinjiang was acquired through landsat5-tm in 2011, and thin cloud was artificially added to the image. The process of simulating adding clouds is as follows: a real thin cloud is added by software to any region of the optical remote sensing image to simulate an optical remote sensing image disturbed by thin cloud.

We compare the proposed method with the following four approaches on optical remote image: the improved homomorphic filtering (IHF) [16], improved retinex (IR) [17], SWT + DWT algorithm (SD) [19] and the Mallat algorithm(M) [20]. To objectively evaluate the processing results, four evaluation indicators were selected in this paper: average gray value, standard deviation, information entropy and average gradient. Among them, the average gray level of the remote sensing image reflects the brightness level of the image. For thin white clouds, the brightness is higher, and the gray level is larger; the standard deviation of the gray level of the image reflects the gray level deviation of the image from each pixel of the image, the degree of grayscale. The details of the image are described. Generally, the larger the standard deviation, the richer the detailed information of the image; the information entropy of the remote sensing image reflects the amount of information contained in the image. A good image for thin cloud removal contains more ground detailed information, so the image content is more abundant, the information entropy is larger, the average gradient of the image represents the contrast degree of the fine part of the image, and the average gradient is larger. The more detailed the contrast, the clearer the image.

As can be seen from Figure 2, compared with IHF and IR algorithms, this paper has obvious advantages in the removal of thin clouds, and the ground object details remain more complete. The results of SD and M algorithms are similar, but the details remain incomplete. It can be seen from Figure 3, compared with the IR algorithm, the algorithm in this paper has a higher degree of cloud removal. Compared with the M algorithm, the algorithm in this paper is better at maintaining ground details, which is conducive to the next step of image processing. In Figure 4, the SD algorithm results are similar to the algorithm in this paper, but the thin cloud removal effect of the algorithm in this paper is more obvious.

### 2.5. Experimental Results and Analysis

As seen from Table 1, the five methods have different degrees of removal of thin cloud noise. Compared with the other four processing methods, the standard deviation of the proposed method is large, and the information entropy value and the standard deviation are large, indicating that the detailed information of the image after removing the thin clouds is relatively large and that the high-frequency ground detail information is maintained or enhanced. The average gradient is improved compared to the improved homomorphic filtering algorithm and the retinex algorithm. The Mallat algorithm is significantly increased, indicating that the detailed contrast is large, and the image is clear. It can be seen that, whether from subjective visual effects or statistical evaluation results, the wavelet transform-based coefficient replacement method proposed in this paper is relatively good for suppressing the thin cloud noise of remote sensing images.

In addition, thin cloud removal experiments on remote sensing images with different resolutions (150 × 150, 500 × 500) in the same area showed that the cloud removal effect of this paper is better than the other four algorithms (IHF, IR, SD and M), which fully illustrates the advantages of the cloud removal algorithm in this paper.

## 3. Image Change Detection Section

### 3.1. Algorithm Description

An unsupervised change detection algorithm based on combination difference graph and FCM clustering is proposed in this paper. To obtain the difference image of the image change information before and after removing the thin clouds, the logarithmic transformation algorithm was first used to transform the image into a logarithmic domain, and the frost filter was used to denoise the image. Then, using the mean ratio method and the difference method, two difference images were obtained. Different denoising methods were applied to the noise characteristics of the two images, and the final difference image was obtained by combining the difference images.

After the thin clouds are removed, we detected the changes of two images. Due to the lack of time-phase diagram data with both thin clouds and contrast images, the measures taken in this paper artificially simulated cloud processing for one of the phase diagrams (time-phase diagram 1 or time-phase diagram 2) during change detection. Therefore, this paper selected 100 time-phase relative ratio telemetry images for artificial simulation and added thin-cloud processing. Specifically, real thin cloud PS (Photoshop) was applied to images that needed change detection. A large number of results show that the objective and subjective evaluation of change detection before and after the removal of thin clouds has significantly improved.

### 3.2. Algorithm Flowchart

**Figure 5:**
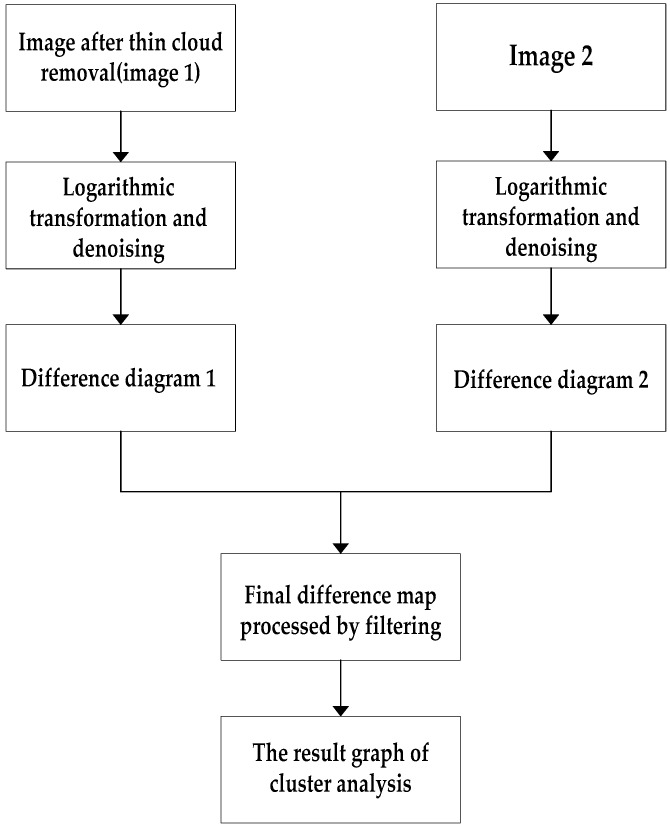
Change detection algorithm.

Figure 5 shows the change detection algorithm.

### 3.3. Experimental Research

**Figure 6:**
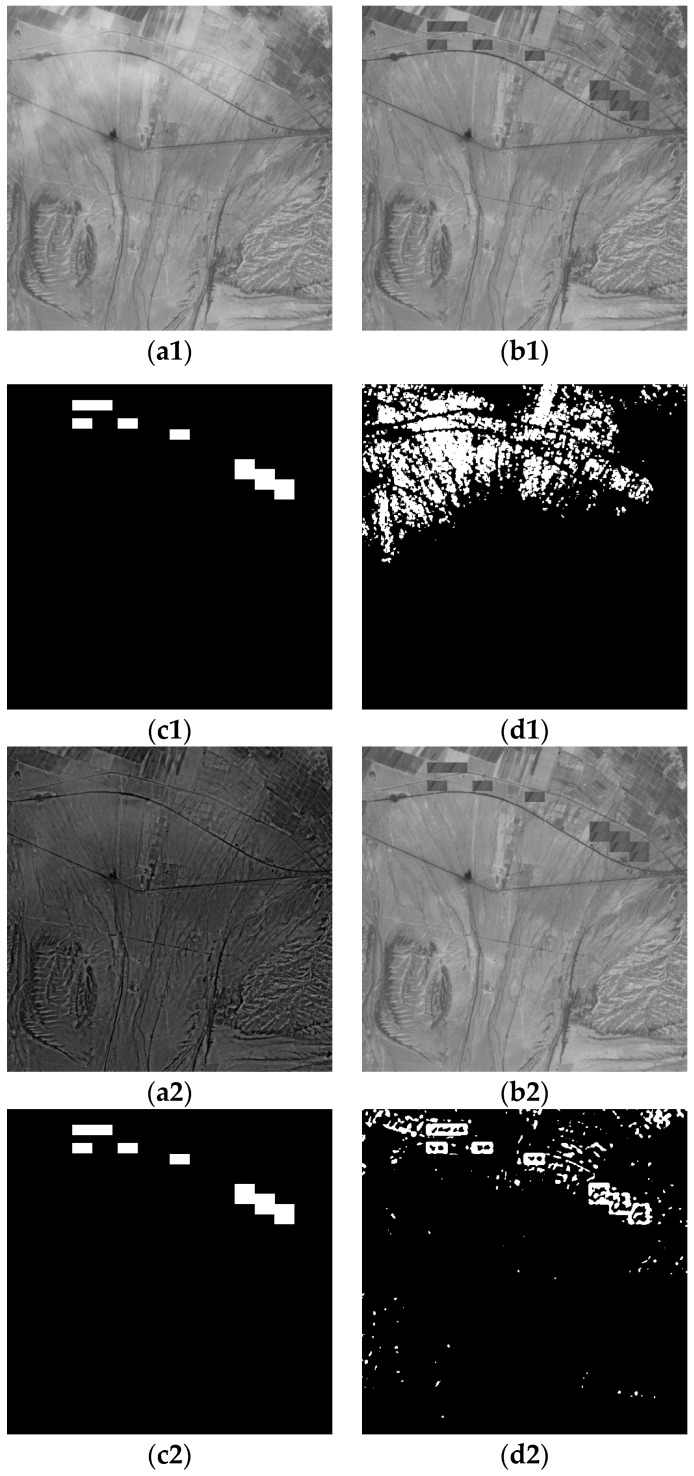
Comparison of changes before and after thin cloud removal: (**a1**) before removing thin clouds (phase one); (**a2**) after removing thin clouds (phase one); (**b1**,**b2**) phase two; (**c1**,**c2**) reference; (**d1**) the results before removing thin clouds; (**d2**) the results after removing thin clouds.

**Figure 7:**
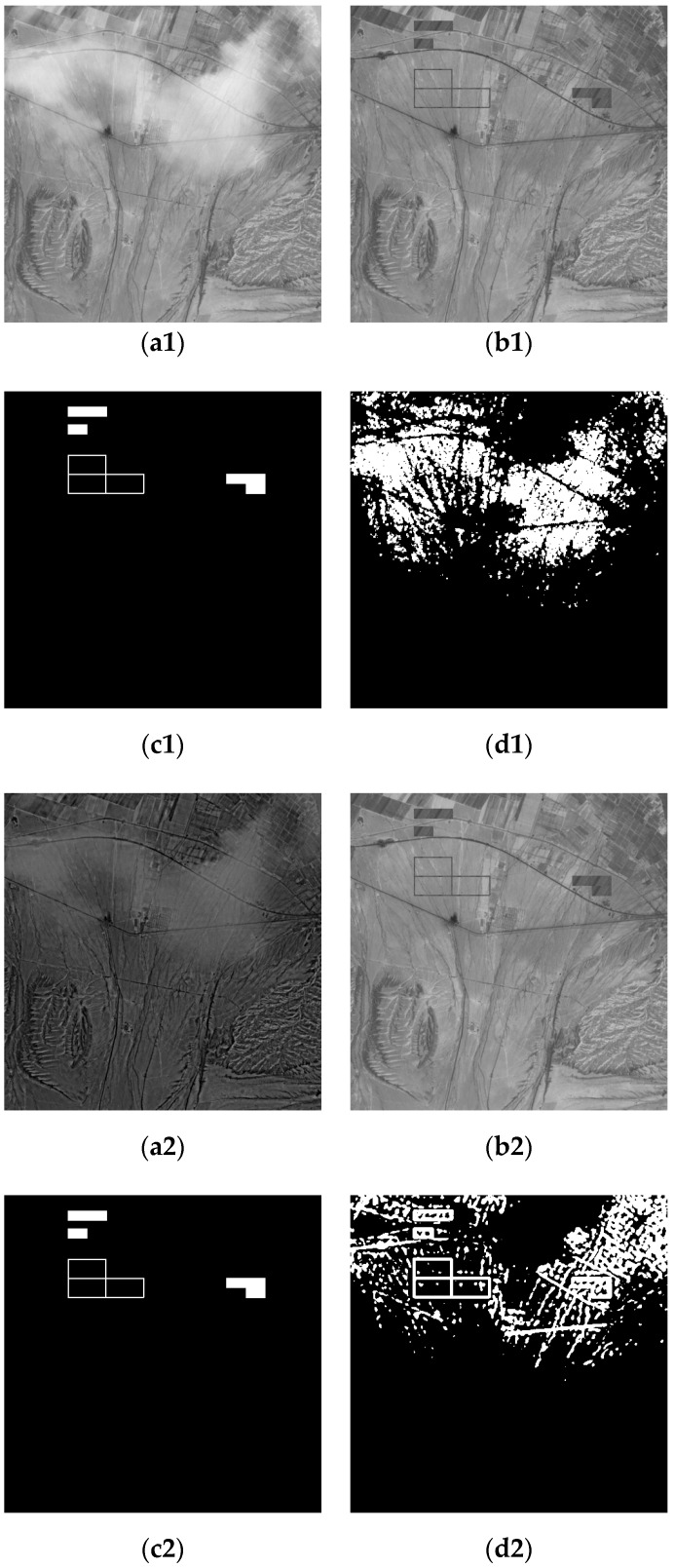
Comparison of changes before and after thin cloud: (**a1**) before removing thin cloud (phase one); (**a2**) after removing thin cloud (phase one); (**b1**,**b2**) phase two; (**c1**,**c2**) reference; (**d1**) the results before removing thin clouds; (**d2**) the results after removing thin clouds.

The experimental data of two groups are the artificial simulation data. In Figure 6 and Figure 7, (a1) is the optical image of Changji region in Xinjiang was taken by Landsat5-tm in 2011, on which thin clouds were artificially added; (b1,b2) are the optical remote sensing image of artificially added change area; (c1,c2) are the change reference image; (d1) is the change detection result under the influence of thin cloud; (a2) is optical image after thin cloud removal; (d2) is the change detection result after removing the thin cloud.

### 3.4. Objective Evaluation Results and Analysis

In this experiment, to evaluate the effectiveness of the proposed algorithm, three objective indicators, overall error, accuracy rate and the kappa coefficient, were used to evaluate the performance of optical remote sensing images before and after thin cloud removal. The results are shown in Table 2. In both sets of experiments, the kappa coefficient and the accuracy rate of the image after removal of the thin clouds increased, and the overall error decreased compared with the image before the removal of the thin clouds. For example, in the detection results of changes before and after cloud removal in Figure 6, compared with those before and after cloud removal, the accuracy rate after cloud removal increased by 9.98%, and the overall error rate decreased by 76%; in Figure 7, the accuracy rate after cloud removal increased by 6.49%, and the overall error rate decreased by 34%.

Compared with the result of change detection of single remote sensing image affected by thin clouds, the experimental results showed that the overall error rate of change detection of two remote sensing images affected by thin clouds increases, the accuracy rate and the Kappa coefficient decrease.

### 3.5. Time Complexity of the Proposed Algorithm

In this part, we will talk about the time complexity of the proposed algorithm. As illustrated in Figure 1 and Figure 5 of the manuscript, the proposed algorithm includes wavelet coefficient replacement algorithm, enhancement algorithm, Frost filtering, DI’generation and FCM clustering. Therefore, the time complexity of the proposed algorithm is the sum of time complexities of these different processes. Let n represent the number of the total pixels in remote images, c is class number of FCM, H_f_ is the number of FCM iteration. The time complexity of each process in the proposed algorithm is shown in the following table. The entire algorithm is O (c2Hf n) as shown in Table 3.

## 4. Conclusions

In order to solve the problem of change detection of optical remote sensing image disturbed by thin cloud, this paper proposed, for the first time, an unsupervised change detection method based on combination difference map and FCM clustering combined with wavelet coefficient substitution algorithm. Firstly, thin clouds are removed from the optical remote sensing images, and then the image change after removing the cloud is detected. In the thin cloud removal part, this paper proposed a new wavelet coefficient substitution algorithm, which combined the characteristics of the thin cloud remote sensing image and enhanced the ground detail information. The experimental results showed that, compared with the objective indicators of the four related algorithms that have been reported so far, the proposed algorithm has achieved better results. In the change detection part, an unsupervised change detection algorithm based on the combination difference map and FCM clustering was used to detect the change of remote sensing images after removing thin clouds. Finally, the change detection experimental results showed that, after removing the clouds from the images disturbed by thin clouds with our proposed algorithm, the effect of optical remote sensing image change detection was significantly improved, so this research direction has a very important practical significance. However, there are still some noises after removing the thin cloud algorithm in this paper, so how to reduce the noise after removing the cloud and improve the accuracy of change detection is our next research work.

## Figures and Tables

**Table 1:** Objective evaluation indicators of remote sensing images before and after thin cloud removal.

Methods	Gray Mean Value	Standard Deviation	Information Entropy	Average Gradient	T/s
Remote sensing images before and after removal of thin clouds (real cloud image 1)					
Original image	91.48	13.78	5.52	0.0141	/
IHF	112.7	41.94	5.45	0.0467	0.86
IR	114.5	44.79	7.392	0.0652	0.61
SD	45.75	10.97	5.171	0.0342	1.36
M	77.19	11.92	5.256	0.0156	1.52
Proposed method	109.66	45.98	7.38	0.097	1.56
Remote sensing images before and after removal of thin clouds (real cloud image 2)					
Original image	69.32	14.48	5.509	0.0117	/
IHF	111.2	35.71	5.509	0.0288	0.77
IR	113.1	42.41	7.233	0.0510	0.52
SD	34.68	9.671	5.106	0.0266	1.29
M	58.92	12.41	5.303	0.117	2.13
Proposed method	104.5	42.81	7.388	0.097	1.82
Remote sensing images before and after removal of thin clouds (artificial simulation to add clouds)					
Original image	144.2	23.85	6.597	0.0153	/
IHF	149.4	34.67	6.597	0.0222	1.16
IR	136.2	56.11	7.788	0.0086	0.62
SD	72.96	16.38	6.049	0.0378	1.47
M	122.5	20.43	6.377	0.0145	1.82
Proposed method	129.4	60.64	7.883	0.0897	2.24

**Table 2:** Image change test results.

**Figure 6**	**Overall Error**	**Accuracy Rate**	**Kappa**
Before removing the thin cloud	32,882	86.85%	0.0538
After removing the thin cloud	7915	96.83%	0.4705
**Figure 7**	**Overall Error**	**Accuracy Rate**	**Kappa**
Before removing the thin cloud	47,294	81.04%	0.0835
After removing the thin cloud	31,129	87.53%	0.1548

**Table 3:** Complexity of each process.

Process	Complexity	Process	Complexity
Wavelet coefficient substitution	O(nlogn)	Enhancement	O(n^2^)
Frost filtering	O(n^2^)	DI’ generation	O(n)
FCM clustering	O(c^2^H_f_ n)	Proposed algorithm	O (c^2^H_f_ n)
